# Age-Related Immunity to Meningococcal Serogroup C Vaccination: An Increase in the Persistence of IgG2 Correlates with a Decrease in the Avidity of IgG

**DOI:** 10.1371/journal.pone.0023497

**Published:** 2011-08-24

**Authors:** Richarda M. de Voer, Fiona R. M. van der Klis, Rutger M. Schepp, Ger T. Rijkers, Elisabeth A. M. Sanders, Guy A. M. Berbers

**Affiliations:** 1 Laboratory for Infectious Diseases and Perinatal Screening, National Institute of Public Health and the Environment, Bilthoven, The Netherlands; 2 Department of Immunology, University Medical Centre, Utrecht, The Netherlands; 3 Laboratory of Medical Microbiology and Immunology, St. Antonius Hospital, Nieuwegein, The Netherlands; 4 Department of Pediatric Immunology and Infectious Diseases, University Medical Centre Utrecht, Utrecht, The Netherlands; University of California Los Angeles, United States of America

## Abstract

**Background:**

All children and adolescents between 1 and 19 years of age in The Netherlands received a single meningococcal serogroup C conjugate (MenCC) vaccine in 2002. During follow-up 4–5 years later, the persistence of MenC polysaccharide-specific IgG was found to be dependent on age of vaccination with higher IgG levels in the oldest immunized age categories.

**Methods and Findings:**

Two cross-sectional population-based serum banks, collected in 1995/1996 and in 2006/2007, were used for this study. We measured MenC polysaccharide-specific IgM, the IgG1 and IgG2 subclasses and determined the avidity of the IgG antibodies. We report that the age-related persistence of IgG after immunization with the MenCC vaccine seemed to result from an increase of IgG2 levels with age, while IgG1 levels remained stable throughout the different age-cohorts. Furthermore, an age-related increase in IgM levels was observed, correlating with the persistence of IgG antibodies with age. It is noteworthy that the increase in IgG2 correlated with a reduced IgG-avidity with age.

**Conclusion:**

These date indicate that the classical characteristics of a T-cell-dependent antibody response as elicited by protein based vaccines might not be completely applicable when conjugate vaccines are administered to older children and adolescents up to 18 years of age. The response elicited by the MenCC vaccine seemed to be more a mixture of both T cell dependent and T cell independent responses in terms of humoral immunological characteristics.

## Introduction

Conjugate vaccines to prevent bacterial meningitis and sepsis caused by pathogens like *Haemophilus influenzae* type B (Hib), *Streptococcus pneumoniae* and *Neisseria meningitidis* have proven to lead to a tremendous decrease in incidence of these diseases when introduced in national immunization programs (NIPs) [1]. Because of the high incidence of diseases in early childhood, particularly in the first 2–3 years of life, vaccination usually needs to start within the first months after birth. However, in contrast to Hib and pneumococcal disease, the incidence of invasive meningococcal infections also shows a second peak in the disease rate among adolescents in the ages 14–19 years [2], [3]. Therefore together with the implementation of meningococcal serogroup C (MenC) immunization in NIPs, many countries also conducted so called catch-up campaigns for children and adolescents up to the age of 24 years [2], [4], [5]. In the Netherlands, a single MenC conjugate (MenCC) immunization (NeisVac-C, Baxter, USA) was implemented in the National Immunization Programme at 14 months of age in 2002 for all newborns and a catch-up campaign was simultaneously initiated targeting all children and adolescents from 1 year up to the age of 18 (vaccine coverage 94%). This approach resulted in an immediate and dramatic decline in MenC disease in all age categories with only few cases in unvaccinated individuals each year without any vaccine failures [6]. This decrease was due to herd effects caused by reduced carriage in the immunized adolescents, who previously had the highest carriage rates [7].

Several serosurveillance studies in a number of countries have been conducted to monitor the persistence of MenC polysaccharide (PS)-specific IgG and serum bactericidal antibodies at different ages after introduction of a MenC conjugate vaccine [8]–[11]. All studies revealed that sustainment of (bactericidal) antibodies after a single MenC conjugate (MenCC) immunization increased with the age at which the vaccine was administered. This is suggested to be due to immune maturation with age and also natural priming with meningococcus during childhood. In the Netherlands, up till 95% of young adults at 22 years who had received a single MenCC vaccine 4–5 years earlier, still had protective antibody levels present [8]. Furthermore, we recently showed that not only antibodies directed towards the polysaccharide gradually increase with age, but also antibodies directed against the carrier protein increased in a similar age-related manner [8]. Unfortunately, data on the development and persistence of vaccine-induced antibodies at increasing age during childhood and adolescence are scarce, and the interval between 2 and 18 years is seldom studied, apart from the rare opportunities given during a catch-up campaign.

In the present study we investigated whether the immune response elicited by the single MenCC vaccine changed with age, not only in terms of height of the antibody levels during childhood and adolescence, but also in terms of type and properties of antibodies induced. We therefore compared two large and unique cross-sectional serosurveillance studies which were conducted in the pre- and post introduction of MenCC vaccination era in the Netherlands [12], [13]. In these cross-sectional cohort studies of persons aged between 0 and 80 years of age we measured MenC-specific IgM levels, as well as the IgG subclass distribution and avidity.

## Materials and Methods

The two cross-sectional serosurveillance studies have been previously described [12], [13]. The pre-MenC introduction serosurveillance study was approved by the medical ethical committee of TNO Prevention and Health in Leiden and performed in 1995/1996. The post-MenC introduction serosurveillance study was approved by the medical ethics testing committee of the foundation of therapeutic evaluation of medicines (METC-STEG) in Almere (clinical trial number: ISRCTN 20164309) and performed in 2006/2007. All participants or parents/guardians of minors involved in both studies provided written informed consent.

### Study samples

The seroprevalences and levels of MenC PS-specific IgG and bactericidal antibodies pre- and post-introduction of the MenCC vaccine in the Netherlands have been described previously [8]. In the present study, MenC PS-specific IgM (*n* = 1096) in different age-cohorts (0–79 years of age) was determined in the same serum sample set from the post-MenC introduction era of which serum bactericidal (SBA) prevalence (*n* = 1220) was previously described [8]. Furthermore, all serum samples from this subset that contained ≥0.25 µg/ml MenC-specific IgG were examined for their meningococcal serogroup A and C-specific IgG1 and IgG2 subclass distribution (*n* = 654) and meningococcal serogroup C PS-specific IgG avidity (*n* = 649). In addition, a set of age-matched serum samples were randomly selected from the pre-MenC introduction serosurveillance study to measure the MenC PS-specific IgM levels in different cohorts (ages 0–79 years) (*n* = 323).

### Detection of MenA and C-specific IgG subclasses and MenC PS-specific IgM

Meningococcal serogroup A and C-specific IgG1, IgG2 and MenC PS-specific IgM antibodies were quantified using a fluorescent-bead-based multiplex immunoassay (MIA) as published before [14], [15]. Standardized reference serum CDC 1992 was used in this assay (NIBSC, Potters Bar, UK). Samples were analyzed using a Bio-Plex 200 system in combination with the Bio-Plex Manager software, version 4.1.1 (Bio-Rad Laboratories, Hercules, CA). For each analyte, median fluorescent intensity was converted to µg/ml by interpolation from the 5-parameter logistic standard curve. The lower limit of quantitation was assigned at 0.01 µg/ml for statistical purposes.

### Analysis of MenC-specific IgG serum avidity

To determine antibody avidity of MenC-specific IgG antibodies a modification of the MIA for measurement of MenC-specific IgG was used, as previously described [16]. Briefly, serum samples with an IgG concentration of ≥0.25 µg/ml were adjusted to an antibody concentration of 25 ng/ml. Ammonium thiocyanate, 0.5M (NH_4_SCN; Sigma-Aldrich, St. Louis, MO) was used to dissociate low-avidity antigen-antibody binding. Samples were measured with the Bio-Plex 200 system as described above. The avidity index (AI) is the percentage of antibodies that remains bound to the MenC PS-conjugated beads after treatment with NH_4_SCN and is calculated as follows: AI  =  (amount of IgG with NH_4_SCN)/(amount of IgG with PBS) ×100. Sera per cohort were subdivided in high, intermediate and low IgG avidity sera, based on the following AI: high, 100–66%; intermediate, 66–33%; and low, 0–33%.

### Statistical analysis

Analysis of the data was performed using GraphPad Prism version 4.00 for Windows (GraphPad Software, San Diego, CA, USA) and SAS 9.1.3 (SAS Institute Inc. Cary, NC, USA). Differences between groups were determined using the non-parametric Mann-Whitney test and correlations were determined using the non-parametric Spearman test. A *P*-value of 0.05 was considered statistically significant.

## Results

### Meningococcal serogroup C-specific IgM

**Figure 1:**
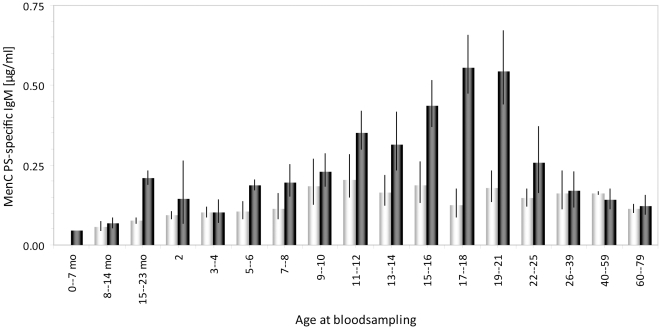
Meningococcal serogroup C PS-specific IgM levels. Levels of IgM in the pre-MenC introduction era are shown in grey bars, the post-MenC introduction era are shown in black bars. Routine immunization is offered in the post-MenC introduction era at 14 months of age and children and adolescents between 5–21 years of age received a single immunization 4–5 years earlier. Error bars indicate 95% confidence intervals. Age at bloodsampling is indicated in years or as stated otherwise (mo  =  age in months).

Previously, we described in a study on adult MenCC immunization, that higher MenC PS-specific IgM levels were present in adults who had received a single MenCC vaccine 5 years earlier as compared to individuals who had not received a MenCC immunization before [16]. In the present study we determined MenC PS-specific IgM levels 5 years after the national MenCC catch-up campaign in different age-cohorts and compared these to levels of IgM in non-immunized aged-matched individuals from the pre-MenC introduction era (Figure 1). A significantly higher level of IgM is observed in the age-cohort to whom the routine infant immunization at 14 months of age is offered as compared to the pre-MenC introduction survey samples (*P*<0.001). However, in the cohorts of children 2 and 3–4 years of age there was already no difference in IgM antibody levels between the pre-MenC and post-MenC group. In line with the previously described IgG levels [8], also a clear age-related increase in persisting IgM levels was observed in samples from the post-MenC introduction survey. IgM-levels in individuals aged 11 to 21 years, that had been immunized 4–5 years earlier, were significantly higher than in the pre-MenC immunization era (*P* < 0.001), although in general still at low concentrations (Figure 1). These higher levels of MenC PS-specific IgM significantly correlated with the higher levels of IgG within the immunized age-cohorts (*r* = 0.7571, cohorts 15 months to 21 years, Table 1). Levels of IgM in the non-immunized age-cohorts (above 25 years of age) were not significantly different between pre- and post-MenC introduction surveys (Figure 1).

**Table 1:** Correlations between levels of IgG, IgG1 or IgG2 and percentages of low or high AI per cohort.

	IgM			IgG1			IgG2			% Low AI			% High AI		
	*r*	*p*	*n*	*r*	*p*	*n*	*r*	*p*	*n*	*r*	*p*	*n*	*r*	*p*	*n*
IgG (cohorts 15 months – 79 year)	0.7571	0.0010	15	0.0750	0.7905	15	0.6964	0.0039	15	0.5571	0.0351	15	−0.7643	0.0009	15
IgG (cohorts 15 months – 21 year)	0.6818	0.0208	11	0.2273	0.5015	11	0.8636	0.0006	11	0.8000	0.0021	11	−0.9727	<.0001	11
IgG1 (cohorts 15 months – 79 year)	0.2893	0.2957	15	ND	ND	NA	−0.5964	0.0189	15	−0.6393	0.0103	15	0.3679	0.1773	15
IgG1 (cohorts 15 months – 21 year)	−0.0909	0.7904	11	ND	ND	NA	−0.1455	0.6696	11	−0.1182	0.7293	11	−0.1909	0.5739	11
IgG2 (cohorts 15 months – 79 year)	0.2536	0.3618	15	ND	ND	NA	ND	ND	NA	0.8786	<.0001	15	−0.8179	0.0002	15
IgG2 (cohorts 15 months – 21 year)	0.9364	<.0001	11	ND	ND	NA	ND	ND	NA	0.8727	0.0005	11	−0.8455	0.0010	11

Spearman's rank correlation coefficient (*r*) with the *p* values (*p*) and the number of cohorts tested for each association (*n*).

ND, not done; NA, not applicable.

### Meningococcal serogroup C polysaccharide-specific IgG avidity

**Figure 2:**
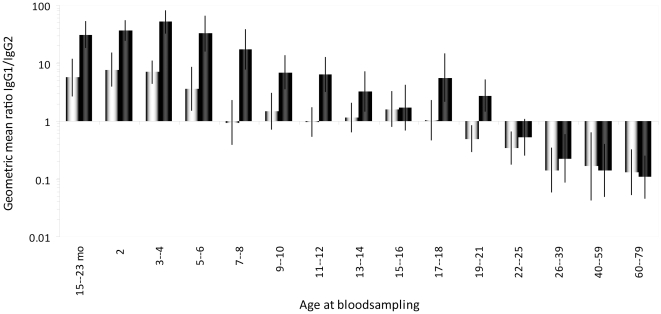
Meningococcal serogroup A and C PS-specific IgG1/IgG2 ratios (grey and black bars, respectively). An IgG1/IgG2 ratio of >1 indicates a higher level of IgG1; an IgG1/IgG2 ratio of <1 indicates a higher level of IgG2. MenCC immunization is offered at 14 months of age and children and adolescents between 5–21 years of age received a single immunization 4–5 years earlier. Error bars indicate 95% confidence intervals. Age at bloodsampling is indicated in years or as stated otherwise (mo  =  age in months).

To see whether the previously observed increase in IgG with age of immunization was linked with increased avidity, which is proposed to be a hallmark of immune memory [17], we determined avidity in various age-cohorts. In age-cohorts who had recently received a single MenCC immunization at 14 months of age during the cohort study in 2006/7, an increase in the fraction of sera with relatively high avidity antibodies was observed (Figure 2). In the cohort 15–23 months, 12% of the sera revealed a relatively high avidity index (AI), which increased up to 56% of the sera at 3–4 years of age. Interestingly, in the catch-up campaign age-cohorts of 2002, the fraction of individuals that showed a relatively high AI gradually decreased with age. In the youngest catch up cohort, (7–8 years of age at time of blood sampling, and 3–4 years at the time of vaccination), 31% of persons revealed a relatively high AI which decreased with age to 14% in the oldest immunized age-cohorts (19–21 years of age at time of blood sampling and 15–17/8 at the time of vaccination) (Figure 2). The percentage of sera with relatively intermediate AI remained rather constant throughout the different vaccinated cohorts. As a consequence of above, the percentage of sera with low AI increased with age. The rise in the percentage of individuals with a relatively low AI positively correlates with the rise of IgG levels with age in the immunized age-cohorts (*r* = 0.8000, Table 1). A negative correlation was found between the percentage of individuals with a relatively high AI and total MenC PS-specific IgG levels in all age-cohorts (Table 1). In non-immunized individuals (above 25 years of age), more than half of all individuals (59%) revealed a relatively low AI.

### Meningococcal serogroup A and C IgG subclass distribution

**Figure 3:**
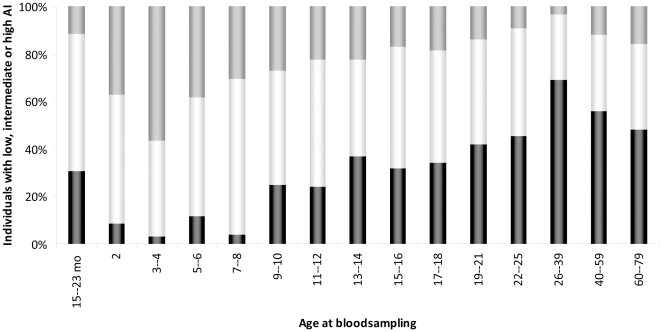
Avidity of Meningococcal serogroup C PS-specific IgG. Sera with an avidity indices (AI) of 0–33 are indicated as low AI (in black), sera with an AI of 34–66 are determined to be of intermediate AI (in white) and sera with an AI of 67–100 are assigned to be of high AI (in grey). Age at bloodsampling is indicated in years or as stated otherwise (mo  =  age in months).

We measured the MenA PS-specific IgG1 and IgG2 levels and IgG1/IgG2 ratio's, which were expected to be elicited by natural exposure or cross-reactivity and not to vaccination [18], and compared these to the MenC PS-specific IgG1 and IgG2 levels and IgG1/IgG2 ratio's from vaccinated cohorts and older non-immunized cohorts in the post-introduction era of the MenCC vaccine (Figure 3). The MenA PS-specific IgG1/IgG2 ratios showed the clear age-specific pattern which is to be expected for TI antigens: an apparent IgG1 response is observed during childhood, illustrated by an on average IgG1/IgG2 ratio of 6 in the cohorts aged 0–6 years, which gradually shifts via a similar IgG1 and IgG2 level in adolescents (average ratio of 1, between 7 and 18 years of age) towards a clear IgG2 response in adults (average ratio of 0.20, above 25 years of age) (Figure 3). The MenC PS-specific IgG1/IgG2 ratios showed a somewhat different picture with age: a significantly higher IgG1/IgG2 ratio (average ratio of 31) is observed in the youngest cohorts (0–6 years) compared to the MenA ratio, which shifts to a clear IgG2 response in the non-immunized cohorts (>25 years of age) with an average IgG1/IgG2 ratio of 0.25 (Figure 3). However, compared to the IgG1/IgG2 ratio of MenA-specific antibodies, higher IgG1/IgG2 ratios in the cohorts between 7 and 21 years of age (average ratio of 6) were observed for MenC. In addition, an inverse correlation between the levels of MenC PS-specific IgG1 and IgG2 within all cohorts (15 months to 79 years of age) was observed (Table 1). However, this correlation does not exist when only the levels of IgG1 and IgG2 in the immunized cohorts were compared (Table 1).

### Correlation between IgG levels, IgG subclass distribution and overall IgG avidity

**Figure 4:**
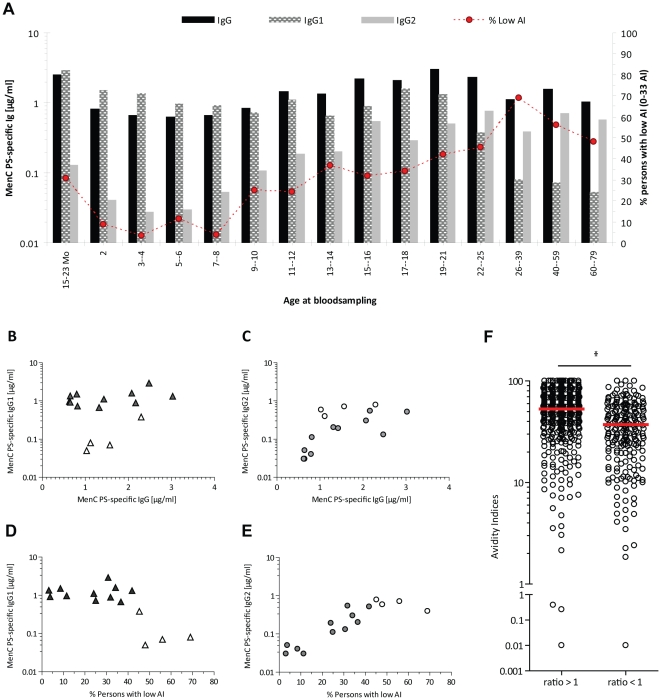
Correlation between levels of Meningococcal serogroup C PS-specific IgG, IgG1, IgG2 and IgG avidity within immunized or non-immunized cohorts in the post-immunization era. A) Concentrations of IgG, IgG1, IgG2 and the percentage of persons with a relatively low AI in each cohort. B) Correlation between IgG1 and IgG in the 11 immunized cohorts (15 months to 21 years of age, filled triangles) or in the 4 non-immunized cohorts (22 to 79 years of age, open triangles). C) Correlation between IgG2 and IgG in the 11 immunized cohorts (15 months to 21 years of age, filled circles) or in the 4 non-immunized cohorts (22 to 79 years of age, open circles). D) Correlation between IgG1 and the percentage of individuals with low AI in the 11 immunized cohorts (15 months to 21 years of age, filled triangles) or in the 4 non-immunized cohorts (22 to 79 years of age, open triangles). E) Correlation between IgG2 and the percentage of individuals with low AI in the 11 immunized cohorts (15 months to 21 years of age, filled triangles) or in the 4 non-immunized cohorts (22 to 79 years of age, open triangles). F) Comparison of the AI's of sera with an IgG1/IgG2 ratio >1 and an IgG1/IgG2 ratio <1. * *P*<0.0001.

Concentrations of IgG, IgG1, IgG2 and the percentage of persons with a relatively low AI in each cohort are depicted in figure 4A. Levels of IgG1 remain at a relative constant level throughout the different immunized cohorts, while the levels of IgG2 rise with the age at immunization (Figure 4A–C). The age-related increase in the prevalence of MenC PS-specific IgG is, besides the correlation with IgM, significantly correlated with the levels of MenC PS-specific IgG2 in all cohorts (*r* = 0.6964). This is further emphasized by an even stronger correlation (*r* = 0.8636) between the levels of IgG with the levels of IgG2 in the immunized cohorts only (cohorts between 15 months and 21 years of age), while there was no significant correlation between the levels of IgG and IgG1 within these immunized cohorts (Figure 4B–C & Table 1). No correlation between levels of IgG, IgG1, IgG2 and serum IgG avidity was found within the non-immunized groups above 25 years of age.

We also found a positive correlation between the increase in IgG2 levels with age and the age-related increase in the percentage of individuals with a relatively low AI in the MenCC immunized cohorts (11 cohorts, age 15 months –21 years), while an inverse correlation existed between the percentage of individuals with low AI and IgG1 levels with age (Figure 4D–E, Table 1). On the other hand, an inverse correlation between the percentage of individuals with a relatively high AI and the increase in IgG2 levels with age was found (Table 1). However, this inverse correlation does not exist when comparing the percentage of individuals with a relatively low AI with the levels of IgG1 within the immunized cohorts only, whereas the positive correlation with IgG2 continues to exist within these cohorts (Table 1). This positive correlation between low AI and IgG2 was even further illustrated when all sera with an IgG1/IgG2 ratio <1 were compared to all sera with an IgG1/IgG2 ratio >1. The sera with an IgG1/IgG2 ratio <1 showed a significantly lower AI than the sera with an IgG1/IgG2 ratio >1 (*P*<0.0001) (Figure 4f). We can therefore conclude that the avidity of MenC PS-specific IgG is mainly determined by IgG1 antibodies. The increase in the levels of IgG2 to MenC with age thus leads to a reduction in the avidity with age.

## Discussion

In the present study we have investigated various aspects of the humoral immune response elicited by immunization with a single MenCC vaccine at different ages 4–5 years after MenCC vaccination and compared these with historic age-matched controls without MenCC vaccination. We found that the age-related levels of MenC PS-specific IgG following vaccination resulted from an increase in IgG2 antibodies with age, while IgG1 remained at a similar level throughout the different age-cohorts. Furthermore, this increase in IgG2 with age was also related to a reduced IgG avidity with age. Besides IgG, also an age-related increase of MenC PS-specific IgM levels was observed, which significantly correlated with the increased levels of total IgG and IgG2 antibodies with age.

Data about the effects of conjugate immunization at older ages compared with infant vaccinations are scarce and are primarily derived from the *H. influenza* type b conjugate vaccine [17], [19], [20]. Our present data from MenCC routine immunized children at 14 months of age show T cell dependent (TD) response characteristics: low levels of IgM, a clear induction of the IgG1 subclass and an increase in avidity of IgG antibodies in the years following immunization, as was previously also seen for other conjugate vaccines [17], [21]. However, our data show that as vaccination occurs at a later age, humoral responses seem to shift towards a T cell independent (TI) response: induction of IgM, increasing levels of IgG2 and a lower avidity of IgG antibodies. Remarkably, the immune response described was measured in a population that was immunized with a single MenCC vaccination 4–5 years earlier. However, a characteristic TI response would have been thought to be transient, with IgG antibody responses that would have declined to low levels within a few years and no induction of immunological memory. Clearly, this is not the case, as memory B cell responses have been demonstrated previously after booster immunization at infant, adolescent and adult ages [16], [22]–[24]. Indeed, if we compare the MenC PS-specific IgG1/IgG2 ratios to the naturally elicited MenA PS subclass ratios in the MenCC immunized cohorts, the major subclass induced by vaccination was found to be IgG1, which should be indicative for a TD response [25]. However, levels of IgG2 increased with age of vaccination and accounted for the increasing prevalence of overall IgG with age. Moreover, we found a reduced IgG avidity with age, while an increase in avidity is considered to be one the main features of a TD response. Low-avidity antibodies predominate during the acute-phase of an immune response, whereas high-avidity antibodies are selected and produced later and thus would have been expected in the years following immunization [26]. In view of this rationale, the presence of low-avidity antibodies was unexpected but might be explained by the higher levels of IgG2 with age, which is in concordance with other studies [27], [28]. Goldblatt et al. found that following MenCC vaccination in adults the already relative high avidity of antibodies failed to mature further in the subsequent 6 months [29]. The index even seemed to decrease slightly in this period which could be in line with our observation of decreasing avidity in the young adults 5 years following their single vaccination. Interestingly, although the antibodies are of low avidity, the amount of antibodies seems to play the major role with respect to protection, since the total levels of IgG correlate well with bactericidal antibodies [8].

Although the increase in levels of IgM observed in the adolescent cohorts may not be of clinical relevance, due to their quite low levels, they do suggest that either a prolonged immune response is initiated or persistent antigen presentation might occur [30]. However, because IgM is considered to be a “natural antibody” [31], the possibility that IgM is elicited by cross-reactive carbohydrates should not be ruled out. This probably might also explain why there are no differences between pre- and post-immunization era levels in the non-immunized individuals, which is in contrast with the IgG levels [8].

Previously, it has been reported that the increased antibody sustainment of MenC-specific antibodies could be caused by natural priming before immunization, immune maturation or possible circulation of the bacterium among the population [10], [32]. Currently, the circulation of MenC in the Netherlands is expected to be very low, reflected by a low disease incidence in non-immunized persons and carriage studies in other countries which have implemented MenCC vacciantion have provided evidence for reduced nasopharyngeal carriage [7]. It is therefore not to be expected that the increased antibody levels observed after introduction of the vaccine are caused by natural exposure to the MenC bacterium. It can of course not be ruled out that other commensal bacteria may cause cross-reactivity [33]. However, we showed earlier that antibodies towards to the carrier protein, tetanus toxoïd, are also maintained in an age-related manner, which makes cross-reactivity less plausible [8]. Sustainment of antibody levels is an active process dependent on both the turnover and synthesis of antibodies [34]. Studies in mice have shown that continuous production of antibodies is dependent on the presence of (persisting) antigen [35], [36]. Whether this is the case when tetanus and MenC PS are administered in a conjugated form is unknown and will be very difficult to demonstrate in humans. However, our current data on persistence of IgM and IgG2 lead to the suggestion that not only maturation and priming of the immune system are implicated in the response towards conjugate vaccines.

As indicated above, the working mechanism of polysaccharide-conjugate vaccines still cannot unequivocally be classified as TD. It has been described that marginal zone B cells play a prominent role in the responses to non-conjugated polysaccharides [37]. This may explain why in infants under 2 years of age immune responses are impaired, because the marginal zone matures around the age of 18 to 24 months [38], [39]. Whether this subset of B cells is also involved in the immune response towards conjugate vaccines is unknown. The induction of immunological memory strongly indicates that follicular B cells are involved and that germinal centres are formed during the primary immune response. However, as Pollard and colleagues describe [3], this would result in a humoral response which is characterized by IgG1, IgG3 and antibodies of high avidity, while we observe in our present study increased levels of IgM, IgG2 and moderate to low avidity. Unfortunately, we were unable to measure the IgG1/IgG2 subclass ratios and avidity in the pre-immunization cohort, since MenC PS-specific antibody levels were very low [8]. Therefore, we cannot exclude that pre-existing MenC IgG levels might have been of influence also [40]. However, the MenA PS-specific antibodies present in adolescent age-groups indicate an equal IgG1 to IgG2 ratio. Therefore, our data may suggest that the immune response towards conjugate vaccines may be comparable but probably not completely identical to the response induced by conventional protein antigens, which involves the subset of follicular B cells, but that perhaps also the “classical” PS-specific subset of marginal zone B cells is involved. Naturally primed memory B cells may possibly participate in this response, since it is likely that exposure to PS during nasopharyngeal carriage may prime B cells, as the PS on the surface of the bacterium may be seen by the immune system as a molecule conjugated to outer-membrane proteins [29].

In conclusion, immunization with the meningococcal serogroup C conjugate vaccine at increasing infant or adolescent age results in increased persistence of IgM and IgG2 and is correlated with the persistence of IgG and reduced IgG avidity. The response to conjugate vaccines at ages above infancy does not seem to be a fully conventional TD response, because it displays characteristics of both TD and TI responses. Further studies should aim at the molecular analysis of B cells and T cells involved in the responses towards conjugate vaccines, which can provide evidence for the nature of cells that are being activated by conjugate vaccines in infants, adolescents and adults.
